# Hearing Loss and Dementia: Where to From Here?

**DOI:** 10.1097/AUD.0000000000001494

**Published:** 2024-02-21

**Authors:** Piers Dawes, Kevin J. Munro

**Affiliations:** 1 Centre for Hearing Research, School of Health and Rehabilitation Sciences, University of Queensland, Queensland, Australia; 2 Manchester Centre for Audiology and Deafness, University of Manchester, UK.

**Keywords:** Dementia, Hearing aids, Hearing loss

## Abstract

Victorian era psychologists were the first to comment on associations between sensory and cognitive function. More recently, hearing loss has been shown as a marker of risk for dementia. However, it is not known whether this association represents a causal impact of hearing loss, nor whether treating hearing loss may help prevent dementia. Most studies on relationships between hearing loss and cognitive outcomes are observational, are at risk of confounding, and cannot reach conclusions about causation. A recent high quality randomized controlled trial, relatively uncommon in audiology, reported no impact of a comprehensive hearing intervention in mitigating cognitive decline in older adults. Although secondary analysis revealed potential benefits in a sub-sample of adults, this finding may be spurious. Encouraging policymakers, patients, and other health care practitioners to address hearing loss in terms of dementia prevention may be inappropriate on the grounds of both relevance at individual level and lack of clear evidence of benefit. In addition, advocating need to address hearing loss in terms of mitigating dementia risk may reduce the importance of addressing hearing loss in its own right. Linking hearing loss to dementia risk may also exacerbate the stigma of hearing loss, inadvertently discouraging people from seeking help for hearing. We suggest that treating hearing loss may have important benefits in preventing or delaying diagnosis of dementia via improving orientation and functioning in daily life, without changing the underlying pathology. Rather than linking hearing loss to dementia risk, we suggest a positive message focusing on the known benefits of addressing hearing loss in terms of improved communication, quality of life, and healthy aging.

## INTRODUCTION

Victorian era psychologists were the first to comment on associations between hearing, vision, and cognitive function (Galton 1883). Some 100 years later, the topic was revisited by researchers in the Berlin aging study. In a sample of people who ranged in age from 25 to 103 years, researchers reported that age-related declines in hearing and vision acuity closely followed declines in cognition, with a strength of association of 0.95 (Lindenberger & Baltes 1994; Baltes & Lindenberger 1997). Intriguingly, the Berlin investigators reported an age-independent association such that variance in sensory function accounted for 12.6% of variance in cognitive function: at any age, people with better sensory function had better cognition. The investigators suggested that this connection between sensory function and cognition might provide a window into cognitive aging, although as discussed later, there could be several explanations for this association. Interest in the connection between sensory function and cognition was given a further boost, when Lin et al. (2011) reported that baseline levels of hearing loss were associated with incident dementia, such that people with more severe hearing loss were more likely to develop dementia over time.

### Links Between Sensory Function and Cognition: Association But Not Necessarily Causation

A systematic review by Loughrey et al. (2018) revealed that associations between hearing loss and lower cognitive function, hearing loss and increased rates of cognitive decline, and hearing loss and dementia risk are well replicated. One interpretation of associations between hearing loss and cognitive outcomes is that hearing loss has a causal impact on cognition. The impact may be direct, via alterations in auditory input impacting on brain structures that support cognition, or indirect, via increased social isolation, reduced self-efficacy, reduced physical activity, or participation in cognitively stimulating activities (Wayne & Johnsrude 2015; Uchida et al. 2019; Griffiths et al. 2020; Völter et al. 2021). However, the causal direction of the association may alternatively be the other way around; that is, cognitive declines impact hearing (Maharani et al. 2020). “Listening” is a highly cognitively challenging activity (McGarrigle et al. 2014; Pichora-Fuller et al. 2016). Someone might experience hearing difficulties due to impaired cognitive function, rather than hearing loss. Lastly, there may be no direct causal link between hearing loss and cognition, but they are associated due to common cause(s) that affect both hearing and cognitive health (Tan et al. 2020), including cardiovascular factors, immune function, and inflammation (D’Amico et al. 2020; Matthews et al. 2022).

A Lancet review on dementia intervention, prevention, and care (Livingston et al. 2020) carried out a synthesis of three studies (Lin 2011; Gallacher et al. 2012; Deal et al. 2017) that linked baseline hearing loss to risk of subsequent dementia. The Lancet review concluded that eliminating or entirely mitigating hearing loss could potentially yield an 8% reduction in the overall number of cases of dementia (Livingston et al. 2020). Although sometimes overlooked or omitted, the inclusion of the word “potentially” is critical because it is not known if the associations do represent a causal impact of hearing loss on cognitive health.

The “8%” statistic does not describe the strength of risk of dementia at an individual level as sometimes stated (Blustein et al. 2020). Rather, this statistic is the “percent attributable fraction.” This fraction combines the strength of each risk factors’ association with incident dementia and the prevalence of each risk within the population (i.e., the levels of exposure to the risk). The percent attributable fraction for hearing loss is relatively high because hearing loss is highly prevalent. In *The Lancet* review, other factors (e.g., depression) had similar size of risk for dementia as hearing loss at individual level. A further caveat is that the percentage attributable fractions reported in *The Lancet* paper pertain to the population risk profiles in the UK and similar high-income countries. Different factors (e.g., smoking; maternal and early life malnutrition) may be more important population-level risks in low- and middle-income countries, as emphasized in *The Lancet* review (Livingston et al. 2020). It is misleading to assert that hearing loss is the leading potentially modifiable risk for dementia unless it is clear that this may only be true for high-income countries. The largest numbers of people living with dementia and the largest projected increases in the numbers of people with dementia are in low- and middle-income countries (Prince et al. 2013).

Studies linking hearing and cognition (including cognitive performance, cognitive decline, and incident dementia) have mostly been observational, with inferences based on patterns of association between variables in population samples. A concerning phenomenon is that these associations are substantially attenuated when demographic and health factors are statistically accounted for. Having “accounted for” these potential confounds, researchers typically conclude that the relatively small remaining association between hearing loss and the cognitive outcome of interest is due to an independent effect of hearing loss on cognition (e.g., Maharani et al. 2018). But the remaining association could alternatively, and parsimoniously, be explained by residual confounding; that is, that there were unmeasured confounds and/or imperfect control of measured confounds.

All factors identified as potentially modifiable risks for dementia (traumatic brain injury, hypertension, alcohol consumption, obesity, smoking, physical inactivity, air pollution, and diabetes) in *The Lancet* review are also known risks for hearing loss. No epidemiological study of associations between hearing loss and dementia and other cognitive outcomes could completely account for these shared risks. However, as *The Lancet* review identified, the appealing prospect is that, if there is a causal impact of hearing loss on cognition, then readily available and relatively low-cost hearing interventions might mitigate the impact of hearing loss on cognition and reduce dementia risk.

### Do Hearing Interventions Reduce Dementia Risk?

The possibility of reducing cognitive decline and mitigating dementia risk has been examined in several observational studies that modeled cognitive outcomes for hearing aid users compared with nonusers (Yeo et al. 2023). In a recent review, we summarized these findings, focusing on studies that examined cognitive outcomes with follow-up greater than 3 years after provision of intervention (Dawes & Völter 2023), selected because this duration may be sufficient to observe the effects of hearing interventions on cognitive decline. We did not identify any studies of cognitive outcomes with non-device-based hearing loss interventions. Non-device-based interventions (e.g., “Active Communication Education”; ACE [Hickson et al. 2007]) are effective in reducing the impacts of hearing loss on communication and psychosocial outcomes, and so may also be effective in improving cognitive outcomes, especially if the impact of hearing loss on dementia risk is via psychosocial pathways (i.e., via depression or social isolation).

In respect to device-based hearing interventions, we examined studies focused on cochlear implants or hearing aids. Because cochlear implants make a bigger difference to hearing than hearing aids, one might hypothesize a bigger effect of cochlear implants on cognitive outcomes. We identified three cochlear implant studies that fit inclusion for our review, all of which reported positive outcomes in relation to long-term cognitive improvements after implantation. These results are encouraging, but the methodological limitations of these studies—including a lack of adequate control group comparison and high attrition rates—preclude any conclusions about the cognitive benefits of cochlear implants (Dawes & Völter 2023).

For hearing aids, the balance of evidence in our review was equivocal. In relation to cognitive impairment (e.g., incident dementia), four studies reported lower incidence for hearing aid users versus nonusers and five reported no difference. In relation to cognitive decline (i.e., changes in cognitive performance over time), four studies reported lower rates of decline and three reported no difference. Thus, a total of eight studies reported positive impacts of hearing aid use and eight did not. These hearing aid studies were all observational (i.e., based on comparing differences in outcomes for hearing aid users versus nonusers in large data sets).

Here again, a challenge with observational studies is the potential for confounding and lack of ability to definitively attribute any positive cognitive outcomes associated with hearing aid users to use of hearing aids. The reason is that only a minority of people with hearing loss use hearing aids, and hearing aid users differ from nonusers in terms of typically being better educated, more affluent, and more likely to be from a majority ethnic background (Sawyer et al. 2019). These demographic factors are related to a wide range of health outcomes, including cognitive ones. If a study reports better cognitive outcomes for hearing aid users versus nonusers, one cannot be sure these better outcomes are due to the hearing aid rather than confounds.

### Prospective Trials of Hearing Aids

Due to the challenges with not being able to definitively attribute any cognitive benefits to hearing aid use, observational studies typically conclude by calling for prospective controlled trials. Randomized allocation to treatment (i.e., hearing aids) or control condition means that potential biases can be minimized, so randomized controlled trials (RCTs) are considered the gold standard in terms of evidence. But trials of the sort that would be needed are challenging. The slow rate of cognitive decline and low rates of incident dementia mean studies need to be very large and long running. One methodological group suggested a minimum sample of 44,000 people would be required to provide sufficient power to detect differences in incident dementia for an intervention trial, assuming participants aged around 70 years (typical of first-time hearing aid users), 4 years of follow-up and a reasonably conservative clinically relevant effect (Richard et al. 2012). So, although dementia prevention is the primary interest, for practical reasons, trialists must focus on cognitive change as the primary outcome of interest. Studies focusing on cognitive change would still need to be relatively large and long-running, but not quite so challenging as those focusing on dementia as the primary outcome. Besides challenges with sample size and study duration, there are scientific challenges with selective dropout and practice effects that bias results. There is also an ethical challenge. Because hearing aids have many proven benefits for people with hearing loss (Ferguson et al. 2017), it is unethical to randomize participants with hearing loss to a control group, withholding the intervention until the end of a long duration study.

We know of only two studies that have grappled with these challenges and conducted RCTs of hearing aids on cognitive change. One is the Cognition and Hearing Loss Project (Jayakody et al. 2020), which is due to report soon. The limitations of this Australian project include the short (for indexing cognitive decline) duration of follow-up of 12 months and the relatively small sample (90 per group).

The other RCT is the Aging and Cognitive Health Evaluation in Elders (ACHIEVE) study, which reported recently (Lin et al. 2023). There are few studies in audiology of comparable scale, potential impact, and rigor; ACHIEVE is aptly named. ACHIEVE randomized 977 adults aged 70 to 84 years with untreated hearing loss to a hearing intervention (audiological counseling, provision of hearing aids and assistive listening devices) or a control condition (health education; chronic disease prevention). The primary outcome was 3-year change in a global cognition standardized factor score from a neurocognitive battery. Participants were recruited either from an observational study of cardiovascular health (the Atherosclerosis Risk in Communities [ARIC] study; n = 238) or recruited de novo from the general community (n = 739). The main finding was no difference in 3-year change in global cognition between the hearing intervention and the control group.

The authors highlighted results from a secondary analysis showing a significant interaction between the ARIC and the de novo cohort, with significant reduction in cognitive decline for the hearing intervention in the ARIC cohort but not in the de novo cohort (but the effect size was small according to Cohen’s d [0.2] and the family-wise error rate does not appear to have been controlled across all the tests presented). Compared with the de novo cohort, the ARIC cohort was older, lower income, had more people with Black ethnic background, higher levels of diabetes, lower baseline cognition, and—oddly—lower hearing aid use among the hearing intervention group. The researchers concluded that “*a hearing intervention might reduce cognitive change over 3 years in populations of older adults at increased risk for cognitive decline but not in populations at decreased risk for cognitive decline.*” (page 1). This was an encouraging conclusion that has been welcomed by the audiology community and garnered much attention. The cover of *The Lancet* in which the ACHIEVE study was published featured the quote “*Based on evidence from the ACHIEVE study, hearing loss might be a particularly important global public health target for dementia prevention efforts*” (cover page, volume 402). In the same volume, Livingston and Costafreda authored a comment on ACHIEVE that noted that the finding of an “*enormous (48%) protective effect of hearing aids on cognition at high-risk population could be spurious*,” but stated that “*hearing aids could really make a difference for populations at risk of dementia*.” (Livingston & Costafreda 2023a). A National Institutes of Health news article headlined “*Hearing aids slow cognitive decline in people at high risk*,” concluded that hearing aids reduce the rate of cognitive decline by almost 50% among older adults at high risk of dementia, and that treating hearing loss may offer a way to lower dementia risk among vulnerable older people (NIH Research Matters 2023). Despite the positive interpretation, some caution may be warranted. The primary reason for caution is that the main finding of ACHIEVE (neglected in the publicity around the trial) was negative. The result from the secondary analysis may be spurious.

### ARIC Benefits: Actual or Spurious?

The finding of a positive effect of hearing aids on cognitive decline in a subgroup of adults at increased risk of cognitive decline is contrary to the epidemiology. Associations between hearing loss and cognitive outcomes, as well as the potential benefits of hearing aids, were observed for general population samples, not only among samples stratified according to high risk of cognitive decline. The ACHIEVE authors, and others, have argued in recent presentations to the audiology community that the de novo sample is not representative of the general population, and that the ARIC results are more generalizable because the ARIC cohort is more like the general population. But a comparison with U.S. population profiles suggests that if anything, the de novo sample is more like the U.S. general population than the ARIC sample, at least in terms of levels of college education, income, proportion of people from Black ethnic background and proportion of men and women (https://www.census.gov/quickfacts/). The finding of a positive effect of hearing aids on cognitive decline in a subgroup of adults at increased risk of cognitive decline also seems contrary to what one might have hypothesized. One would have thought that the effect of hearing aids would be greatest in those with few other risks for cognitive decline besides hearing loss. Hearing aids are surely most effective at mitigating the impact of hearing loss and would likely have much less effect in mitigating other risks for cognitive decline (e.g., diabetes). A subgroup of people with many other risks for cognitive decline would be less amenable to cognitive decline-altering treatment with a hearing-focused intervention. Following good research practice, the ACHIEVE protocol was published before the study was completed (Deal et al. 2018). The protocol paper reported planned examination of interactions between intervention condition and race, study site, baseline cognition, baseline hearing loss and recruitment source (ARIC or de novo). But it did not report any hypotheses concerning different effects of the intervention in the ARIC or de novo subgroups. Another finding that raises questions about the reliability of an effect of the hearing intervention on cognitive decline was that there was no interaction between the hearing intervention and hearing loss severity (pure-tone average [PTA] <40 dB versus ≥40 dB) on cognitive decline. One would have thought that any effect of hearing intervention would be greater among those with more severe hearing loss.

### Spoken Cognitive Tests

ACHIEVE included a battery of 10 neurocognitive tests. Two were reported to be exclusively auditory, the remaining eight had visual stimuli or both auditory and visual stimuli. Effects of hearing loss vary according to the demands of individual cognitive tests, but in general, hearing loss is known to affect performance on cognitive tests that include spoken elements (Füllgrabe 2020). ACHIEVE did not report on any immediate improvements in cognitive test performance after provision of hearing aids (which would indicate an effect of improved audibility) nor outcomes broken down according to tests with a visual-only component compared with those with auditory components. It is unclear from the peer-reviewed article whether any benefits of the hearing intervention for the ARIC group may have been due to participants being able to hear the cognitive test stimuli better. As the levels of hearing loss seem similar across the ARIC and de novo subgroups, one explanation could be that improved audibility was especially important for the ARIC subgroup, because those in the ARIC subgroup had lower baseline levels of cognition and were less able to compensate for the impact of hearing loss on performance of verbally presented cognitive tests (McGarrigle et al. 2014).

### Hearing Intervention: Generalizability

ACHIEVE provided an impressively comprehensive hearing intervention that included provision of bilateral hearing aids and other assistive hearing devices (e.g., streamers and remote microphones) provided free of charge to participants via four 1-hr sessions with an audiologist every 1 to 3 weeks after fitting. The sessions included systematic orientation and instruction in device use as well as self-management and communication strategies. Participants received booster visits every 6 months over the 3 years of the trial. The ACHIEVE intervention is more comprehensive and intensive than is typically provided in usual clinical services, at least in terms of what we are familiar with in Australia and the UK. There were relatively high levels of hearing aid use (average 7 to 8 hr per day) and low levels of nonuse (10 drop-outs out of 588) in ACHIEVE compared with levels of hearing aid use reported in surveys of people who obtained hearing aids through standard care pathways (Dillon et al. 2020). If there were an effect of the hearing intervention in ACHIEVE, it is not clear that this effect would generalize to more typical hearing aid services or if services have the capacity to expand current intervention models.

According to descriptions of the ACHIEVE sample (Huang et al. 2023), 30% of participants had mild hearing loss (better-ear PTA4 30 to 34.9 dB HL) while 70% of the participants had moderate or greater hearing loss (better-ear PTA4 of 35 to 69.9 dB HL). Moderate or greater hearing loss is much less prevalent than mild hearing loss (Humes 2023) which may also limit the generalization of study findings (although note that there was no interaction of the intervention with hearing loss severity, as discussed earlier).

### Healthy Aging Control Condition May Have Same Cognitive Benefits as Hearing Intervention

To mitigate ethical concerns about withholding a beneficial intervention and to provide an active control condition, the ACHIEVE study used a healthy aging intervention—the 10 keys to Healthy Aging programmer. Ten keys focuses on lifestyle changes to address chronic disease risk factors. Controlled evaluations reported that 10 keys were effective in terms of improved low-density lipoprotein levels, cholesterol, blood pressure control, and blood glucose control in diabetics. Ten keys also promoted uptake of colon cancer screenings, higher levels of physical activity, and better hypertension management (Newman et al. 2010). The 10 keys intervention is much like Finnish Geriatric Intervention Study to Prevent Cognitive Impairment and Disability (FINGER), a multidomain intervention involving diet, exercise, cognitive training, and vascular risk monitoring aimed at reducing cognitive decline in older people. Now replicated in numerous countries, FINGER is effective at reducing cognitive decline (Ngandu et al. 2015). Given the similarity between 10 keys and FINGER, it might be that in ACHIEVE, a hearing intervention was just as effective as a healthy lifestyle intervention in reducing cognitive decline. Unfortunately, the study design is not informative about whether both 10 keys and the hearing intervention were effective (or ineffective) in reducing cognitive decline.

ACHIEVE is an impressive and important study that supplied additional gold-standard evidence for the benefits of hearing aids in reducing hearing handicap. But despite many strengths, ACHIEVE did not supply convincing evidence for the benefits of hearing interventions on cognitive decline as hoped, especially by the audiology community. Like all good research, ACHIEVE raised further questions—some of which may be answered in subsequent analyses to emerge from the trial (e.g., elucidating mechanisms of action for the potential benefits of the hearing intervention observed for the ARIC subgroup).

### Where to From Here?

#### Emphasize the Benefits of Treating Hearing Loss for Its Own Sake

Traditionally, hearing loss has been viewed by clinicians and the public alike as something that is a natural part of aging, mild, innocuous, and unimportant (Armitage et al. 2021). In actual fact, hearing loss is one of the largest sources of burden in terms of years lived with disability (Mathers & Loncar 2006) and a major public health challenge. Invariably, almost any research study on hearing loss published today will provide a background for the research by saying something like “hearing loss is important because hearing loss is risk for dementia” (including papers that we have co-authored, e.g. (Allum, Meredith, Uus, Kirkham, & Dawes, 2023; Dawes et al., 2015; Taylor, Dawes, Kapadia, Shryane, & Norman, 2023)). Implicit in these statements is the idea that addressing dementia is more important than addressing hearing loss. We may risk confirming the assumption that hearing loss is not important per se if we justify a need to address hearing loss in terms of mitigating risk of dementia.

There may also be a risk that other healthcare practitioners, policymakers, and funders of research and clinical services may lose interest in hearing—an important component of healthy ageing—if the publicity given to links between hearing loss and dementia risk and benefits of hearing interventions in preventing dementia is shown to lack a firm foundation. As an example, a compelling study was published in *The Lancet Public Health* journal (Jiang et al. 2023). On the basis of a rigorous analysis of the very large UK Biobank dataset, it reported that hearing aid use was associated with a substantial reduction in risk of all-cause dementia compared with people with self-reported hearing loss who did not use hearing aids. *The Lancet Public Health* paper received a lot of publicity and attention, as well as generating two subsequent editorials extolling the results, adjuring attention to hearing loss, and exhorting the potential to address hearing loss to reduce risk of dementia (Livingston & Costafreda 2023b; The Lancet Public Health 2023). Unfortunately, the original paper in *The Lancet Public Health* journal and the subsequent editorials were quietly retracted when an error in the analysis was discovered: the codes for hearing aid users and nonusers were mixed up (Retraction Watch 2024). In fact, risk of dementia was higher for hearing aid users than non-hearing aid users. This finding is plausible because in the UK Biobank, hearing aid use is a reliable phenotype for hearing loss (Wells et al. 2019), and hearing loss is a well-replicated marker of risk for all-cause dementia (Loughrey et al. 2018). It remains to be seen if the revised findings and interpretation will be reported. It is an on-going struggle, but audiologists should not lose faith in the need to raise awareness of the prevalence and impact of hearing loss in terms of its impact on communication and quality of life. Hearing loss is worth addressing in its own right.

#### Reinforcing Stigma of Hearing Loss

With good intentions, hearing aid manufacturers and audiologists have an understandable wish to motivate people to seek help for hearing loss by linking hearing loss to risk of dementia (Blustein et al. 2020). But identifying hearing loss as a risk for dementia may actually make people less likely to seek help for hearing loss (Fairchild et al. 2018). Scare tactics have often been used as public health strategies (e.g., in relation to smoking cessation or the AIDS epidemic) to encourage desired health behaviors. But some health behavioral research suggests that scare tactics may actually make people less likely to engage in the message being communicated (Fairchild et al. 2018). Linking hearing loss to dementia risks reinforcing the stigma and resulting sense of personal threat, denial and dis-engagement with hearing care that some people may experience in relation to hearing loss diagnosis (Wallhagen 2010; Blustein et al. 2023). Positive messaging about the known benefits of hearing aids in improving communication and promoting an active, socially engaged lifestyle may be more effective in promoting uptake of hearing interventions.

An additional concern is that, if there is a benefit of hearing aids in preventing dementia, it is likely small and probably not clinically significant at the individual level, even though hearing intervention may translate to substantial numbers of cases of dementia prevented at population level. In our view, it is therefore inappropriate to promote dementia prevention as a key benefit of hearing aids on the grounds of both relevance at individual level and lack of clear evidence for such a benefit.

### The Nexus Between Hearing, Cognition, and Functioning in Daily Life

**Fig. 1.**
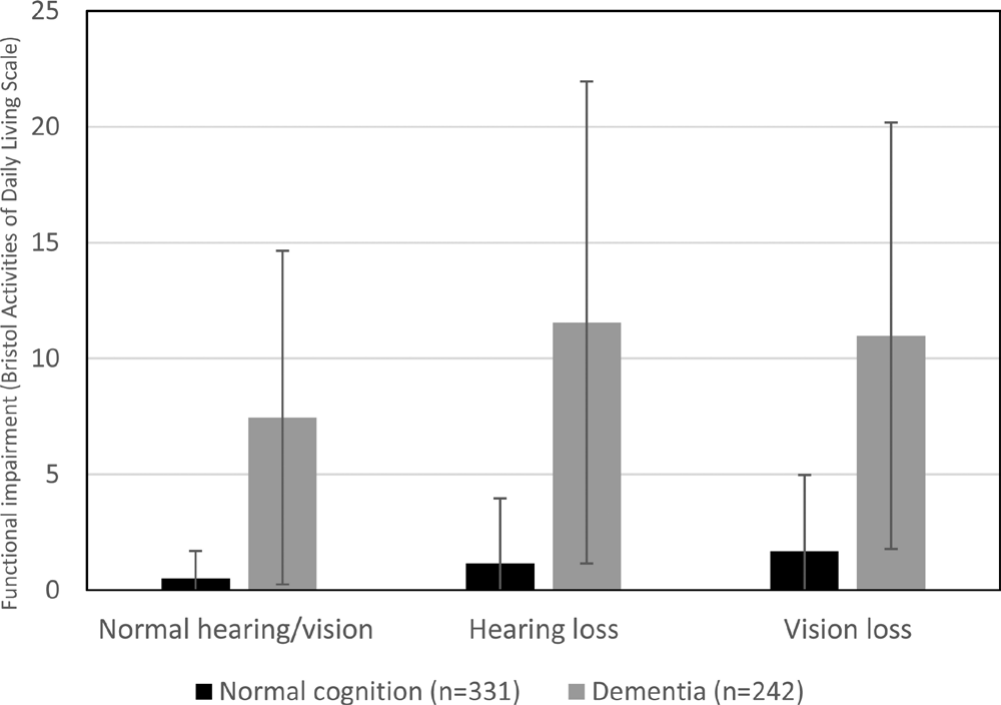
Interaction of hearing loss with cognitive impairment on functional ability. Error bars show SD.

Given the main findings of the ACHIEVE trial, it is unlikely that hearing aids are effective at reducing cognitive decline among people with hearing loss. But although hearing aids may not affect the underlying pathology of dementia and the progress of cognitive decline, hearing aids may reduce risk of dementia by supporting independent function in daily life. Hearing loss results in activity limitations in communication and orientation that affect ability to function in daily life. Hearing loss may also interact with cognitive impairment to exacerbate functional impairments. In a survey of European adults with healthy cognition or dementia and either hearing or vision impairment, we found that comorbid sensory impairment resulted in excess functional impairment among those with cognitive impairment (as revealed by the higher grey bars in the visual and hearing impaired groups in Fig. 1; unpublished data from a trial of hearing and vision interventions for people with dementia [Leroi et al. 2017, 2020]).

The effect of hearing loss on performance is well recognized. In a study of the impact of hearing loss on performance on a memory task, Rabbitt wrote “…*a wide range of other behavioral difficulties in the elderly may often be misattributed to central changes when they are the consequence of mild, and easily remediable, peripheral sensory changes*.” (Rabbitt 1991) (p. 167). The major benefit of hearing aids—well established in several RCTs—is in reducing hearing disability and functional benefits in communication and orientation. This nexus between hearing, cognition, and functioning in daily life is probably where the main benefits of hearing aids lie in terms of preventing or delaying dementia. This possibility could be tested by assessing functional improvements following hearing aid provision and subsequent rates of dementia diagnoses, or alternatively, by seeing whether some people diagnosed with dementia still meet criteria for that diagnosis once their hearing loss is addressed (Littlejohn et al. 2020).

### Supporting Hearing Needs of People With Dementia

Because both hearing loss and dementia are strongly age associated, hearing loss and dementia are commonly comorbid (Kricos 2006). One survey of people living with dementia in the general community reported 87% having clinically significant hearing loss (Allen et al. 2003). Hearing loss may be even more common among people in long-term care settings (Weinstein 1986; Cohen-Mansfield & Taylor 2004). Most cases of hearing loss go unrecognized and untreated among people with dementia (Allen et al. 2003; Nirmalasari et al. 2017). High rates of untreated hearing loss are a particular concern among people with dementia, because hearing loss exacerbates the impact of dementia on quality of life, mental well-being, social participation, communication, independence, and carer burden (Resnick et al. 1997; Dawes et al. 2018; Mamo et al. 2018). Untreated hearing loss also exacerbates symptoms associated with dementia including aggression, hallucinations, and agitation (Haque et al. 2012; El Haj et al. 2017). Timely identification and treatment of hearing loss offers an opportunity to improve these outcomes (Dawes et al. 2018; Mamo et al. 2018; Cross et al. 2022), and improving quality of life for people living with dementia is a global priority (Livingston et al. 2020). However, hearing professionals report lacking confidence in working with people with dementia (Wright et al. 2014; Leroi et al. 2019), or think that people with dementia would not be able to use of benefit from hearing interventions. But reliable hearing assessment is possible, if the assessment is adapted for the needs of people with dementia (Dawes et al. 2022) and systematic reviews show that people with dementia do benefit from hearing interventions (Dawes et al. 2018; Mamo et al. 2018; Cross et al. 2022). Excessive attention to the possibility of hearing interventions preventing dementia may detract from much needed attention to timely identification and treatment of hearing loss for people living with dementia. Current hearing service arrangements may not be well suited to the needs of people with dementia (Dawes et al. 2022). Audiology must strive to be a dementia-friendly profession, with audiologists able to recognize when a patient has dementia, and to provide appropriate patient-centered care to insure optimal quality of life for people living with dementia.

## CONCLUSIONS

Given the imperative to optimize function and quality of life with global population aging and the prominence of hearing loss in the global burden of disease analysis (Mathers & Loncar 2006), it is timely that hearing care professionals engage with stakeholders to improve outcomes for people with hearing loss. For hearing professionals and the public, key messages are around raising awareness of the prevalence and impact of hearing loss on quality of life, and the proven benefits of hearing interventions in facilitating an active, independent, engaged, and healthy older age. Focus should be on the proven benefits of hearing interventions rather than negative messaging about associations between hearing loss and dementia risk. For health care practitioners, key messages could be around holistic care including hearing needs and the importance of communication in healthcare delivery and quality of care. For policymakers, we should emphasize the benefits of hearing interventions in facilitating healthy aging, maintaining independent functioning, and reducing health care costs.

When we ask “where to from here?,” we ask because we are confronted with a problem yet to be solved in a satisfactory way. Leveraging concerns about dementia may not provide the solution, but continued collective effort on multiple fronts may yet do so.

## ACKNOWLEDGMENTS

K.J.M. is supported by the NIHR Manchester Biomedical Research Centre. Grant number: NIHR203308.
